# Warfarin-Capped Gold Nanoparticles: Synthesis, Cytotoxicity, and Cellular Uptake

**DOI:** 10.3390/molecules24224145

**Published:** 2019-11-15

**Authors:** Loredana Florina Leopold, Dumitriţa Rugină, Ioana Oprea, Zorița Diaconeasa, Nicolae Leopold, Maria Suciu, Vasile Coman, Dan Cristian Vodnar, Adela Pintea, Cristina Coman

**Affiliations:** 1 Faculty of Food Science and Technology, University of Agricultural Sciences and Veterinary Medicine, Mănăștur 3-5, 400372 Cluj-Napoca, Romania; loredana.leopold@usamvcluj.ro (L.F.L.); ioana.oprea@usamvcluj.ro (I.O.); zorita.sconta@usamvcluj.ro (Z.D.); dan.vodnar@usamvcluj.ro (D.C.V.); 2 Faculty of Veterinary Medicine, University of Agricultural Sciences and Veterinary Medicine, Mănăștur 3-5, 400372 Cluj-Napoca, Romania; dumitrita.rugina@usamvcluj.ro (D.R.); apintea@usamvcluj.ro (A.P.); 3 Faculty of Physics, Babeș-Bolyai University, Kogălniceanu 1, 400084 Cluj-Napoca, Romania; nicolae.leopold@phys.ubbcluj.ro; 4 Electron Microscopy Center, Faculty of Biology and Geology, Babeș-Bolyai University, Clinicilor 5-7, 400006 Cluj-Napoca, Romania; maria.suciu@itim-cj.ro; 5 National Institute for Research and Development of Isotopic and Molecular Technologies, Donath 67-103, 400293 Cluj-Napoca, Romania; 6 Institute of Life Sciences, University of Agricultural Sciences and Veterinary Medicine, Mănăștur 3-5, 400372 Cluj-Napoca, Romania; vasile.coman@usamvcluj.ro

**Keywords:** gold nanoparticles, D407 cells, HFL-1 cells, internalization

## Abstract

Currently, research studies on nanoparticle cytotoxicity, uptake or internalization into the body’s cells are of great interest for the improvement of diagnostic and therapeutic applications. We report here the synthesis and characterization of very stable novel warfarin-capped gold nanoparticles with an average diameter of 54 ± 10 nm which were prepared using sodium warfarin as a reducing agent. The nanoparticles were tested in terms of cytotoxicity and cellular internalization in vitro on two cell lines: normal lung fibroblast HFL-1 and human retinal pigment epithelial D407 cells. Our results showed that the normal lung fibroblast HFL-1 cells were more sensitive to the nanoparticle treatment compared to the human retinal pigment epithelial D407 cells. Moreover, any signs of potential cytotoxicity occurred during the first 24 h of treatment, the cellular viability remaining largely unchanged for longer exposure times. Transmission electron microscopy and dark field hyperspectral imaging revealed that the nanoparticles were effectively delivered and released to the HFL-1 and D407 cells’ cytoplasm. Our results provide valuable information to further investigate sodium warfarin-capped gold nanoparticles for possible biological applications.

## 1. Introduction

The high interest in metal nanoparticles stems from their quantum scale dimensions, generally below 100 nm, that lead to unique electronic, optical, and magnetic properties and thus their various applications in the biomedical, nanobiotechnology, materials science, chemistry, and physics fields [1,2]. As a consequence of their unique properties, gold nanoparticles (AuNPs) find multiple applications in nanomedicine including diagnostics and imaging [3,4,5], drug therapy, gene delivery, photothermal cancer therapy [6,7,8,9,10,11,12,13,14,15], and the newly developed field of theranostics [16,17].

Gold nanoparticles can be synthesized in a large variety of shapes and sizes. Their electronic, optical, and chemical properties are strongly affected by their size, shape, degree of aggregation, and surface chemistry [4,18,19,20], aspects that can also influence their cellular uptake and cytotoxicity. As opposed to bulk gold, which is essentially inert, higher reactivity can arise at the nanoscale and, therefore, possible associated health and toxicity risks as well. Despite the continuously increasing number of studies focusing on the in vitro and in vivo toxicity assessment of AuNPs, there is still concern and uncertainty regarding their safety and toxicity [6,21].

Several methods have been reported for the synthesis of AuNPs [22]. The most common protocols are based on sodium citrate [23] and sodium borohydride reduction [24], leading to spherical AuNPs with diameters between 10 and 150 nm. Seeding is specially used for obtaining anisotropic particles [25,26,27]. Many other preparation methods have been reported for the synthesis of colloidal gold, using various reducers [4,28,29,30,31], which generate particles with various geometries and sizes. Understanding the nanoparticles’ biocompatibility and internalization in cells is of great interest for diagnostics and therapy.

Here, we report the synthesis of AuNPs capped with warfarin (a well-known anticoagulant) [32], their cytotoxicity, and internalization in two normal cell lines: human fetal lung fibroblast (HFL-1) and human retinal pigment epithelial (D407) cells. The obtained AuNPs had a polygonal shape, mean size of 54 ± 10 nm measured with transmission electron microscopy (TEM), and showed characteristic surface plasmon resonance bands at 540 nm. The cellular internalization was investigated by TEM for both cell lines and by dark field hyperspectral imaging for the D407 cells. The gold nanoparticles were able to cross the cellular membrane and were mainly localized in lysosomes or free in the cell cytoplasm. According to MTT viability assays, the normal lung fibroblast HFL-1 cells were more sensitive to the warfarin-capped AuNPs treatment compared to the human retinal pigment epithelial D407 cells and any potential cytotoxicity occurred during the first 24 h of treatment.

## 2. Results and Discussion

### 2.1. AuNPs Characterization

**Figure 1:**
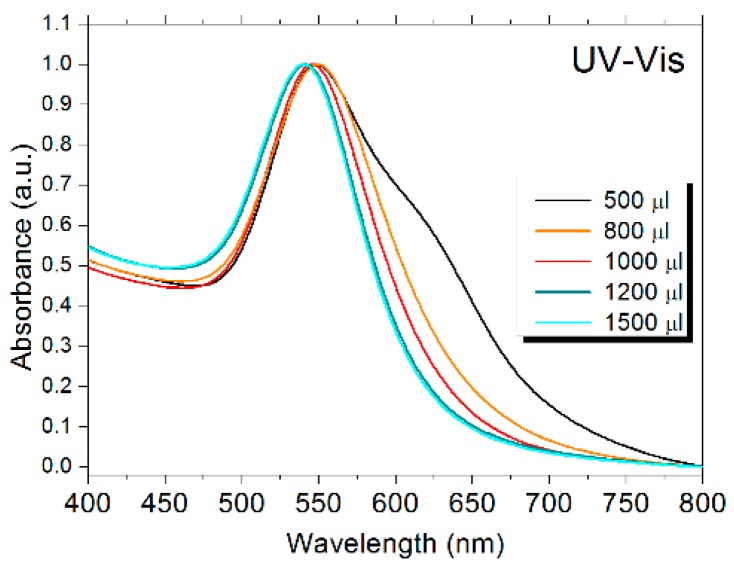
Normalized UV-Vis absorption spectra of the AuNPs obtained by reducing the HAuCl_4_ salt with different amounts of 1% aqueous warfarin solution.

The reduction of the HAuCl_4_ salt and the formation of the AuNPs were confirmed by UV-Vis spectroscopy measurements (Figure 1).

Addition of 1% warfarin solution to the gold salt created changes in color, from colorless to different tones of pink purple. The color of the colloidal AuNPs solutions and the wavelength of the surface plasmon resonance peak depends on the nanoparticles size, geometry, and monodispersity of the samples [6]. The wavelength of the surface plasmon resonance peaks decreased with an increase in the amount of warfarin in the reduction process. The lowest wavelength of the surface plasmon resonance peaks implied the smallest nanoparticle size as the reduction process became complete. The reason for the shoulder at approximately 625 nm when adding low warfarin amounts (500 μL) was that the reduction process of Au^+3^ was far from being completed and the nanoparticles were probably more anisotropic and not uniform in shape. The appearance of the shoulder in the plasmonic band right after the synthesis started (addition of warfarin) was not surprising. Todor et al. (2014) [33] observed a more pronounced shift of the plasmonic band during the synthesis process. At saturation, narrow peaks were obtained with absorption maxima at 540 nm. This was basically the point where enough excess of the reducing agent was added so that the Au was totally reduced, and the appearance of the UV-Vis spectra did not change with the increase in the amount of reducing agent.

**Figure 2:**
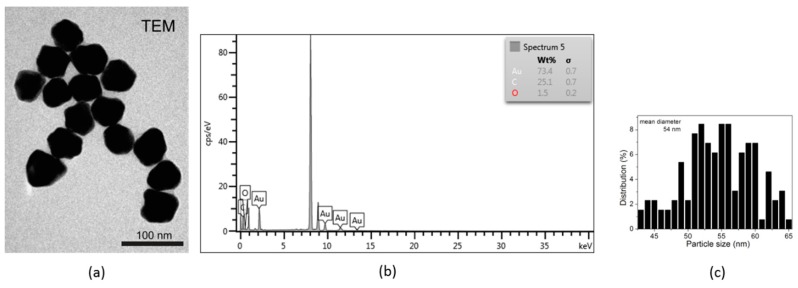
(**a**) TEM micrographs of the colloidal AuNPs (scale bar 100 nm); (**b**) EDS spectrum of the AuNPs; (**c**) particle size distribution from TEM.

A representative TEM image of the AuNPs is shown in Figure 2a. The image shows polygonal nanoparticles, predominantly hexagonal and with a mean size of 54 ± 10 nm. Our values were very close to the previously reported ones, and the slight difference in absorbance most likely came from the polygonal geometry of the warfarin-reduced AuNPs compared to the citrate-reduced ones reported by Haiss et al. [34]. The size distribution histogram of the AuNPs, as obtained from the analysis of the TEM micrographs, is illustrated in Figure 2c, showing size variations in the 43–65 nm range. Elemental analysis was also performed by energy-dispersive spectroscopy (EDS). The characteristic spectrum of the nanoparticles is shown in Figure 2b. The characteristic peaks prove the presence of elemental Au in the samples, as well as C and O which were expected due to the presence of the warfarin capping layer onto the nanoparticle surface.

**Figure 3:**
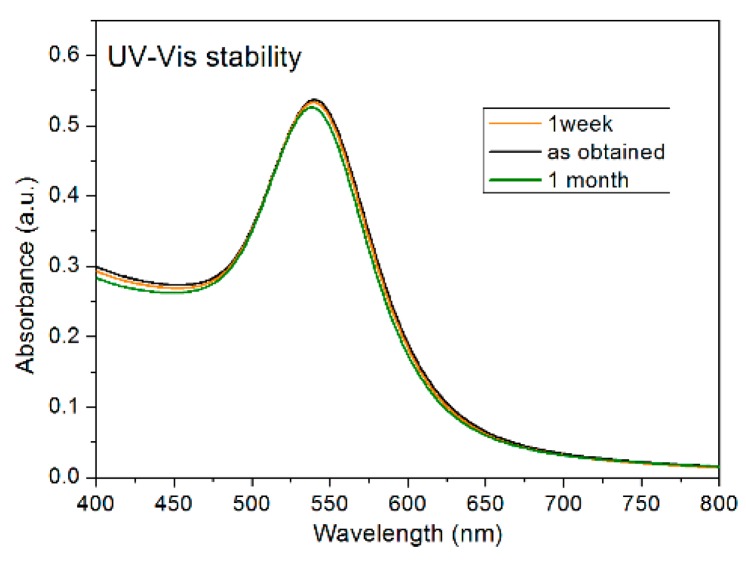
UV-Vis spectra of the AuNPs after one week and one month from the synthesis process.

Since the stability of a colloidal solution is related to the surface charge of the particles, the stability of the prepared colloidal nanoparticle solution was assessed by zeta potential measurements. The measured zeta potential of the AuNPs was −37.7 ± 4.1 mV. This indicates the presence of negatively charged particles with good stability due to the electrostatic repulsions among the nanoparticles. Another proof of nanoparticle stability comes from the analysis of the UV-Vis spectra in time, at one week and one month post-synthesis (Figure 3). The appearance of the spectra was very similar, proving, once again, good stability.

**Figure 4:**
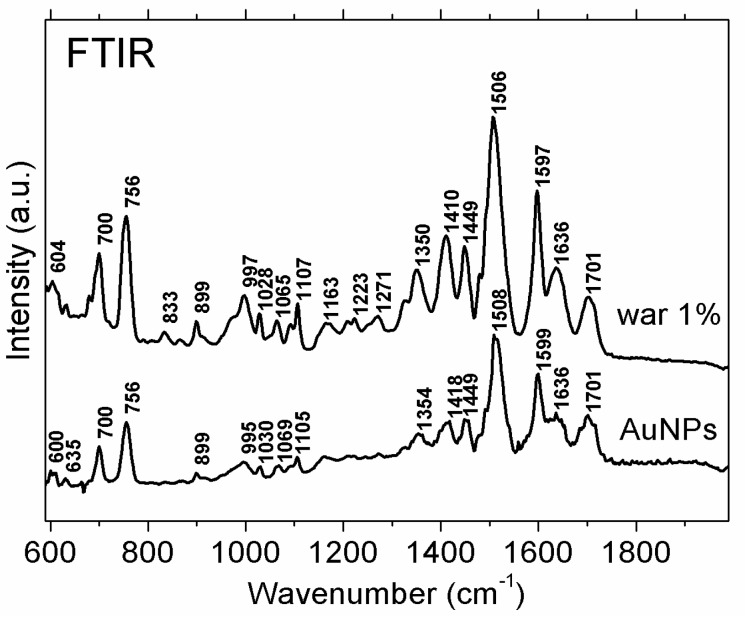
FTIR spectra of the colloidal AuNPs obtained by reducing the HAuCl_4_ salt with warfarin (bottom spectrum) and a 1% warfarin solution in water (upper spectrum).

Figure 4 shows typical FTIR spectra for the colloidal AuNPs (bottom spectrum) and for an aqueous 1% warfarin solution (upper spectrum). Prior to the FTIR experiments, the colloidal samples were centrifuged and washed three times to remove excess reagents. The spectra showed the same characteristic peaks, indicating that the nanoparticles were capped with warfarin, being thus both the reducing and capping agent. The stretching vibrations of the two C=O bonds of warfarin were observed as a large band at 1701 cm^−1^. The bands at 1597 cm^−1^ and 1506 cm^−1^ can be assigned to C=C stretching in the warfarin solution. The asymmetric and symmetric bending vibrations of the CH_3_ group were observed at 1449 cm^−1^ and 1350 cm^−1^, respectively. Further, the out-of-plane bending vibrations of C–H of the phenyl rings were observed at 899 cm^−1^, 756 cm^−1^, and 700 cm^−1^. The band at 1223 cm^−1^ can be assigned to the hemiketal ring in-plane bending vibration. For the nanoparticles capped with warfarin, vibrational bands with lower intensities compared to the warfarin reference spectrum were observed.

### 2.2. Cytotoxicity and Cellular Uptake

**Figure 5:**
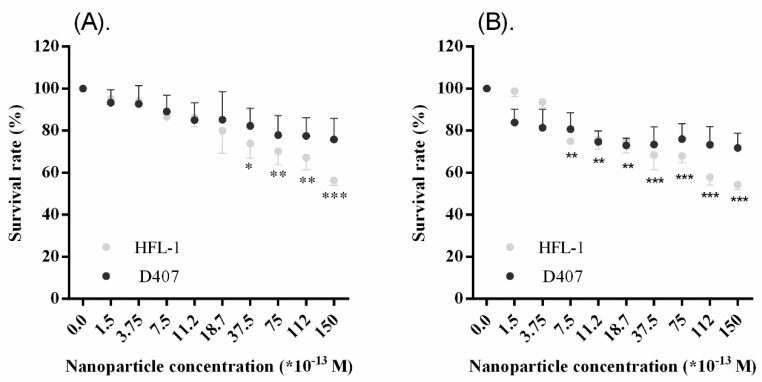
Cell viability assessed by MTT assay (mean ± SD; *n* = 3). The AuNPs’ effect on the survival rate of HFL-1 and D407 cells after 24 h (**A**) and 48 h (**B**) of treatment. Statistically significant differences: (∗ = significant, *p*-value range 0.01–0.05, ∗∗ = very significant, *p*-value range 0.001–0.01, and ∗∗∗ = extremely significant, *p* < 0.001).

Toxicity experiments were performed using HFL-1 and D407 cell lines after incubation with warfarin-capped gold nanoparticles for 24 h and 48 h in complete DMEM at different particle concentrations (up to 150 × 10^−13^ M). For the whole concentration range tested, no significant changes in cell survival rate were observed by increasing the exposure time to nanoparticles from 24 to 48 h, indicating that any potential cytotoxic effects are expected to be observed at a maximum within the first 24 h of exposure, more likely during the first hours. Independent of the duration of the nanoparticle treatment, nanoparticles administered in lower concentrations (0–37.5 × 10^−13^ M) did not have any significant deviation from the control (untreated cells with completed DMEM). However, when administrating higher concentrations, a dose-dependent reduction was observed, and it must be noted that HFL-1 proved to be more sensitive than the D407 cell line (Figure 5). The survival rate in both cases was reduced to a maximum 20% for D407 cells and 40% for HFL cell line, respectively.

### 2.3. Internalization and Cellular Localization of AuNPs

**Figure 6:**
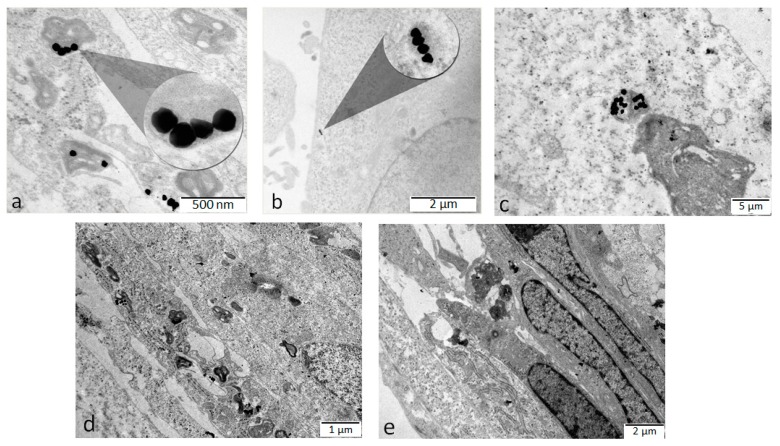
TEM micrographs obtained from (**a**,**d**,**e**) HFL-1 and (**b**,**c**) D407 cells treated by 75 × 10^−13^ M AuNPs for 24 h. The images show the intracellular distribution of the AuNPs in the cells. All the observed intracellular AuNPs were either clustered inside lysosomes or cytosol compartments. Scale bars: (**a**) 500 nm, (**b**) 2 μm, (**c**) 5 μm, (**d**) 1 μm, (**e**) 2 μm.

The warfarin-capped gold nanoparticles were successfully internalized into HFL-1 and D407 cells as observed on microtome sections analyzed by TEM (Figure 6).

Transmission electron microscopyoffers a very high spatial resolution owing to the small de Broglie wavelength of electrons [35]. On both cell lines, internalized warfarin-capped gold nanoparticles were visible as densely packed clusters confined in myelinic-like lysosomes. Also, electron-dense areas of AuNPs located in the cell cytoplasm were observed, indicating the presence of non-entrapped, isolated nanoparticles. These nanoparticles were not included in endosomal/lysosomal vesicles and were rather free in the cytoplasm. Most probably, the internalization mechanism was an endocytic one, in which nanoparticles were enclosed in endosomes, then fused into lysosomes, and, finally, some of the particles were released in the cytosol [36]. This last situation is the most desirable for potential drug delivery systems based on gold nanoparticles. The main limitation of the delivery techniques based on nanoparticles described so far is the ultimate vesicular localization [19,21,37,38,39] of nanoparticles and their unavailability in the cytosol or in the cell nucleus as free particles ready to exert their desired health effects. As other studies report [37,40,41], most of these nanoparticles have the tendency to aggregate in clusters inside cells, which is also the case here. As seen from the lower magnification TEM micrographs in Figure 6d,e, the HFL-1 cells were 5–7 µm long cells, with elongated nuclei, one large electron-dense nucleolus, and an extensive endoplasmic reticulum. Ribosomes can clearly be seen aligned along the endoplasmic reticulum and spread in the cytoplasm.

### 2.4. CytoViva Enhanced Dark Field Hyperspectral Imaging

Thanks to its unique, patented illumination optics, darkfield hyperspectral imaging provides detailed maps of surface plasmon resonance spectra for every pixel. It is possible to image nanoparticles in cells with very good resolution. Another great advantage of the system is that large surface areas corresponding to large numbers of cells can be investigated in a short time as opposed to TEM which requires time-consuming embedding and sectioning steps.

The CytoViva enhanced dark field hyperspectral imaging instrument combines dark field microscopy with NIR-Vis hyperspectral imaging, making it possible to obtain optical imaging and spectral characterization of nanoparticle samples present in biological environments such as cells and tissues [42,43]. The system does not require the use of any fluorescent labels, and the resolution can go down to 2 nm.

**Figure 7:**
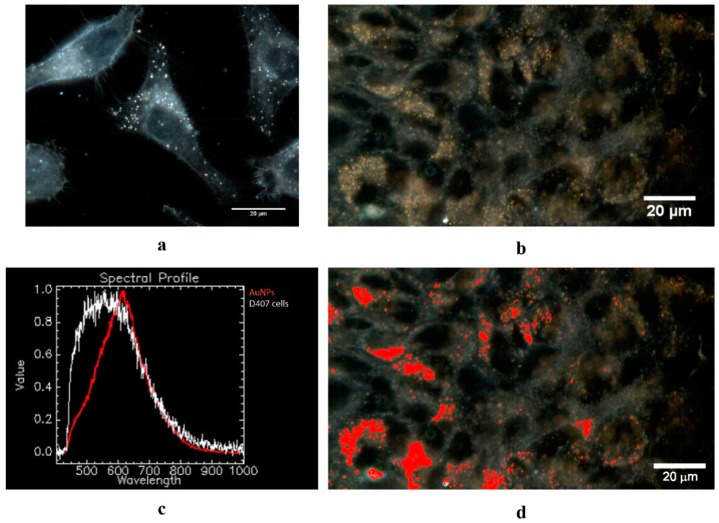
(**a**) Enhanced dark field hyperspectral images of control D407 cells. Scale bar: 20 nm, magnification 100×. (**b**) Hyperspectral image of AuNPs within D407 cells; (**c**) spectral profile of AuNPs and D407 cells; (**d**) AuNP spectra (red) mapped in D407 cells.

Figure 7a is an enhanced darkfield hyperspectral image of a control D407 sample. The darker areas represent the nuclei of the cells, while the bright spots represent endosomes within the D407 cells. In Figure 7b, an enhanced dark field hyperspectral image of D407 cells treated with AuNPs (75 × 10^−13^ M) is shown. The AuNPs cause a different spectral response in areas where they are present in the cell cytoplasm. A comparison between the spectral fingerprint of warfarin-capped nanoparticles in cells (red) and the spectral response of the D407 cells (white) is shown in Figure 7c. The differences in the spectral fingerprint of the warfarin-capped nanoparticles and cells allows a spectral mapping of the sample, making it possible to distinguish the warfarin-capped nanoparticles inside cells. In Figure 7d, the AuNPs spectra are mapped in the D407 cells. The pixel areas representative for the AuNPs in the cells are shown in red in order to clearly distinguish their presence in the D407 cell cytoplasm. The AuNPs were mostly present as aggregates, but areas of dispersed nanoparticles were also present as well.

## 3. Materials and Methods

### 3.1. Chemicals and Materials

Hydrogen tetrachloroaurate(III) (HAuCl_4_*4H_2_O) was purchased from Merck (Darmstadt, Germany). Warfarin sodium (3-(α-Acetonylbenzyl)-4-hydroxycoumarin sodium salt) was purchased from Aldrich (St Louis, MO, USA). Dulbecco’s modified Eagle’s medium (DMEM), fetal bovine serum (FBS), and 3-(4,5-dimethylthiazol-2-yl)-2,5-diphenyltetrazolium bromide (MTT) were purchased from Lonza Group Ltd. (Basel, Switzerland). Kaighn’s Modification of Ham’s F-12 Medium was purchased from GIBCO (Cralsbad, CA, USA). The mixture of penicillin and streptomycin was purchased from Sigma Chemical Co. (St. Louis, MO, USA). Glutaraldehyde, OsO4, Epon 812 resin, and copper grids were purchased from Agar Sientific (Essex, UK); phosphate buffer components and acetone were purchased from Sigma.

### 3.2. Cell Cultures

The human fetal lung fibroblast HFL-1 cell line was purchased from American Type Culture Collection (ATCC) (Manassas, VA, USA). According to the ATCC’s recommendation, it was maintained in Kaighn’s Modification of Ham’s F-12 Medium (F-12K) with fetal bovine serum (10%), glutamine (200 mM), and antibiotics (1%). The human retinal epithelial D407 cell line was kindly donated by Horst A. Diehl, University of Bremen. The D407 cells were cultured in high glucose DMEM, containing fetal bovine serum (10%), glutamine (200 mM), and antibiotics (1%). Both cell lines were maintained and cultivated in standard conditions of 37 °C, 5% CO_2_, and a relative humidity of 95%.

### 3.3. Nanoparticle Synthesis

For the synthesis of AuNPs, different amounts of warfarin 1% solution (500–1500 μL) were added to 50 mL of 0.1 mM HAuCl4 at boiling and the solution was further boiled for 5 min. The protocol resulted in the formation of purple-red colloidal solutions with pH 8.

### 3.4. Characterization Methods and Zeta Potential

The UV-Vis spectra of the AuNP colloidal solutions were recorded on a Perkin Elmer Lambda 25 spectrometer (Perkin Elmer, Waltham, MA, USA) using 1 cm path length quartz cuvettes. The spectral resolution was 1 nm, and the spectra were recorded at 400–800 nm. The FTIR spectra were recorded in the attenuated total reflectance (FTIR-ATR) mode using a Shimadzu IR-Prestige FTIR Spectrometer (Schimadzu, Tokio, Japan) equipped with a diamond PIKE MIRacle single-reflection plate unit. The measurements were performed with a resolution of 4 cm^−1^ in the wavenumber range of 600–4000 cm^−1^, and each spectrum was obtained by co-addition of 64 interferograms. For the FTIR-ATR spectra recordings, drops of colloidal AuNP solution were placed on the ATR plate and dried. For removal of excess reagents from the synthesis process, the as-obtained samples were centrifuged three times at 13,000 RPM for 10 min.

The TEM experiments were carried out using a JEOL model JEM1010 (Jeol, Peabody, MA, USA) transmission electron microscope equipped with a MegaViewIII CCD camera, operating at 100 kV accelerating voltage. For the morphology characterization of the nanoparticles, a drop of colloidal AuNP solution was placed onto a copper grid and allowed to dry. The average size of the AuNPs was estimated using the ImageJ software (version 1.46, national Institues of Health – NIH, Bethesda, MD, USA).

The zeta potential was measured using a Malvern Zetasizer Nano ZS equipped with a 4 mW He–Ne laser operating at 633 nm and an avalanche photodiode detector (Malvern Instruments, Worcestershire UK). Zeta potential was measured by laser Doppler electrophoresis. Three replicate measurements were carried out at 25 °C.

### 3.5. Cellular Internalization

#### 3.5.1. TEM

For visualization of cellular internalization by TEM, cells HFL-1 (1 × 10^3^/well) and D407 (8 × 10^3^/well) were seeded for 24 h on Falcon cell culture inserts (0.4 μm) (Becton Dickinson Labware, NJ, USA). Afterwards, the cells were incubated for another 24 h with 75 × 10^−13^ M AuNPs. The culture medium was then removed, and the cells were washed three times with phosphate buffer saline (PBS) and prefixed for 1 h with glutaraldehyde (2.5% in PBS). Next, the three steps of rinsing with PBS and cells were post-fixed for 1 h in osmium tetroxide (2% in PBS). Dehydration was carried out in HPLC grade acetone in distilled water dilutions, followed by embedding with Epon resin. The resin was polymerized at 60 °C for 48 h then cooled for 12 h. Ultra-thin sections of approximately 70 nm, obtained on a diamond knife (Diatome) with Leica UC6 ultramicrotome, were post-stained with lead citrate and uranyl acetate [35]. Sections collected on 200 mesh Cu grids were examined with TEM.

#### 3.5.2. CytoViva Dark Field Hyperspectral Imaging

The D407 and HFL-1 cells (8 × 10^4^ cells/chamber) were seeded on sterile glass slides for 24 h. Next, 75 × 10^−13^ M AuNPs were applied on both cell lines for another 24 h. After that, the fixation procedure was carried out after three PBS washing steps by incubating cells with 4% paraformaldehyde for 20 min at room temperature. Cells without nanoparticle treatment were used as control. A CytoViva Enhanced Dark Field Hyperspectral Microscope (CytoViva Inc., Auburn, AL, USA) mounted on an Olympus BX-43 optical microscope was used to image the treated and non-treated D407 and HFL-1 cells. The system contains a patented illumination optics that improves up to 10 times the signal-to-noise ratio, making it possible to visualize nanoparticles (down to ~10 nm). The images were taken using the 100× and 60× (UPL Fluorite) oil immersion objectives.

### 3.6. Cytotoxicity Assay

Cell viability was measured by the MTT assay. The MTT detection principle is based on the reduction of the MTT reagent to colored formazan by mitochondrial and cytosol dehydrogenases, which can be detected by measuring its absorbance at 550 nm. The HFL-1 and D407 cells were seeded separately in 96 well plates at a concentration of 5 × 10^3^/well and 8 × 10^3^/well, respectively. After 24 h from seeding, the cells were treated with different nanoparticle concentrations (1.5–150 × 10^−13^ M). Cell viability was monitored 24 h and 48 h post-treatment. Briefly, cells were washed with PBS and then incubated with MTT (0.5 mg/mL) for 1 h. After that the formazan crystals were solubilized in dimethyl sulfoxide (DMSO), their absorbance was monitored at 550 nm and 630 nm (for the background) using the microplate reader HT BioTek Synergy (BioTek Instruments, USA). The results were expressed as the survival rate compared to control. Each experiment was repeated three times.

### 3.7. Statistical Analysis

Statistical analysis was performed using the Duncan multiple comparison test in GraphPad Prism version 5.00. Registered data represented the mean values and standard deviations (SDs) of three experiments (ns = not significant, *p* ≥ 0.05, ∗ = significant, *p*-value 0.01–0.05, ∗∗ = very significant, *p*-value 0.001–0.01, and ∗∗∗ = extremely significant, *p* < 0.001).

## 4. Conclusions

In this study we obtained stable, polygonal-shaped AuNPs using sodium warfarin. The AuNPs were able to cross the cellular membrane of the HFL-1 and D407 cells. After entering the cells, the nanoparticles were visualized being localized in the cytoplasm, mainly in lysosome compartments; however, free nanoparticles were also present. A dose-dependent reduction in cell viability was observed upon exposure to higher concentrations of warfarin-capped nanoparticles in both D407 and HFL-1 cells. The survival rate was reduced by a maximum 20% for D407 cells and 40% for the HFL cell line. It must be noted that HFL-1 proved to be more sensitive than the D407 cell line to the warfarin-capped nanoparticle treatment. Also, exposing the cells to nanoparticles for 48 h did not induce any significant changes in the cell survival rate compared to the 24 h exposure. By using the dark field hyperspectral imaging, it was possible to image the warfarin-capped nanoparticles’ location in cells and to obtain, at the same time, spectral confirmation of their presence. The obtained nanoparticles may be of interest for potential biological applications.
